# Evaluation of Gram-negative bacterial infection by a stable and conjugative bioluminescence plasmid in a mouse model

**DOI:** 10.1186/s12929-014-0078-y

**Published:** 2014-08-19

**Authors:** Yao-Kuang Huang, Chishih Chu, Chih-Hsiung Wu, Chyi-Liang Chen, Cheng-Hsun Chiu

**Affiliations:** 1 Graduate Institute of Clinical Medicine, College of Medicine, Taipei Medical University, Taipei, Taiwan; 2 Division of Thoracic and Cardiovascular Surgery, Chang Gung Memorial Hospital, Chiayi, Taiwan; 3 Department of Microbiology, Immunology, and Biopharmaceutics, National Chiayi University, Chiayi, Taiwan; 4 Molecular Infectious Disease Research Center, Department of Medical Research, Chang Gung Memorial Hospital, No. 5, Fu-Hsin Street, Kweishan, Taoyuan, Taiwan; 5 Division of Pediatric Infectious Diseases, Department of Pediatrics, Chang Gung Children’s Hospital, and College of Medicine, Chang Gung University, No. 5, Fu-Hsin Street, Kweishan, Taoyuan, Taiwan

**Keywords:** Conjugative plasmi, Plasmid stability, Bioluminescence, Mutagenesis, Gram-negative bacteria

## Abstract

**Background:**

The green fluorescence protein (GFP)-associated fluorescence method and the luciferase-associated bioluminescence method are the two major methods for IVIS imaging system to investigate the bacterial infection in animal models. The aim of this study was to evaluate the infection route of Gram-negative bacteria carrying a stable and broad range of conjugative bioluminescence plasmid pSE-Lux1 in a mouse model.

**Results:**

Both encapsulated and non-encapsulated Gram-negative bacteria were used as hosts to evaluate conjugation efficiency and plasmid stability of pSE-Lux1, a recombinant of pSE34 and *luxABCDE* operon*.* The plasmid conjugation efficiencies of pSE-Lux1 ranged from 10^−3^ to 10^−7^ in various Gram-negative bacteria. Plasmid pSE-Lux1 maintained in *Escherichia coli*, *Klebsiella pneumoniae*, and *Salmonella enterica* serovars Choleraesues (abbreviated *S*. Choleraesuis) and Typhimurium (*S.* Typhimurium), than in *Acinetobacter baumannii* and *Serratia marcescens*, was shown to be of better stability for at least four days. To investigate systemic bacterial infections, *K. pneumoniae* strain CG354 was intravenously injected, and then was clearly observed to be non-pathogenic to Balb/c mice for a long-term bioluminescence monitoring for 6 days. For examining dynamic distributions of gastrointestinal tract infection, the invasion protein SipB-deficient mutant OU5045△*sipB* and OU5046△*sipB* of *S.* serovar Typhimurium constructed in this study, compared to wild-type strain OU5045 and its virulence plasmid-less strain OU5046, were of less virulence to mice.

**Conclusions:**

This is the first study to evaluate the conjugative and stable bioluminescence vehicle system of pSE-Lux1 in a wide range of Gram-negative bacteria, a system that can provide a useful reporter approach to trace systemic and gastrointestinal bacterial infections in a mouse model.

## Background

Traditional *in vivo* animal models to investigate microbiologic infection require sacrifice for tissue smear and culture. Although numerous methods have been developed to image bacteria, the clinical applications are still limited due to inefficient *in vivo* detections for a long term of observation without additions of selective antibiotics and substrates, such as lucifirins [1]. Recently, bioluminescence expression system has been developed to trace microorganisms in a living animal [2]. Bioluminescence system in live bacteria is regulated by a *luxABCDE* operon, in which *luxAB* genes encodes for luciferases and *luxCDE* genes encode lipid acid reductases to catalyze the reduction of long chain lipid acids into aldehyde compounds that are the substrates specific for the catalysation of luciferases LuxA and LuxB [3].

Recently, many *luxABCDE*-carrying vectors have been constructed for bioluminescence assays; however, a steady, broad-host auto-bioluminescence vehicle is needed, especially a conjugatable plasmid is preferred to deliver exogenous DNA between bacteria and overcome the difficulty in transformation for some thick-capsule bacteria, *such as Klebsiella pneumoniae*[2],[4]*.* Earlier, we constructed a conjugative vehicle pSE-Lux1, which is a chimeric vector in combination of a stable conjugative native pSE34 that include *pilX1*, *pilX2*, *pilX4*, *pilX5*, *pilX6*, *pilX7*, *pilX8*, *pilX9*, *pilX10*, *pilX11*, *taxA*, *taxB*, and *tax* of type IV secretion system, with a bioluminescence reporter p3ZLux4 that contains the *luxABCDE-kan* operon of pXen-5 (Bioware, Caliper Life Sciences, USA) and the portion of pGEM®-3Z (Promega Corporation, USA) with 300–400 copies per bacterial cell [5]–[8]. Although pSE34 carries *pir*, *parGF* and *stbDE* for plasmid stability and partition, plasmid stability of pSE34 has not been evaluated [7].

In this study, the bacterial conjugation efficiency and plasmid stability of pSE-Lux1 were determined in various Gram-negative bacteria, and distributions of the pSE-Lux1-carrying bacteria in Balb/c mice were evaluated by an *in vivo* imaging system.

## Methods

### Bacterial strains

The Gram-negative bacteria and plasmids used in this study are listed in Table 1. Bacteria were routinely incubated with Luria-Bertani (LB) media (Difco™, Becton Dickinson, USA). *Salmonella* serovars were verified by the O- and H-antigen agglutination tests (Difco™). Plasmid was checked using the alkaline lysis method [9]. Antimicrobial agents ampicillin (100 μl/ml), chloramphenicol (30 μl/ml), gentamicin (50 μl/ml), and kanamycin (50 μl/ml) were used for bacterial selection.

**Table 1:** **Gram-negative bacterial species and plasmids used in this study**^**#**^**Ap**^**r**^**: ampicillin resistance; Cm**^**r**^**: chloramphenicol resistance; Gm**^**r**^**: gentamicin resistance; Km**^**r**^**: kanamycin resistance; Str**^**r**^**: streptomycin resistance**

**Strains and plasmids**	**Characteristics**^**#**^	**Note**
*E. coli* TOP10	Δ*lacX74 ara*Δ*139*Δ*(ara-leu)*	Invitrogen®
*Salmonella* Typhimurium		
OU5045	Wild type with a virulence plasmid	Ou and Chu [10]
OU5046	A virulence plasmidless strains derived from OU5045	Ou and Chu [10]
OU5045△*sipB*	OU5045 with *sipB* deletion; Cm^r^	This study
OU5046△*sipB*	OU5046 with *sipB* deletion ; Cm^r^	This study
*Salmonella* Choleraesuis OU7085	Clinical isolate; drug-susceptible	Ou and Chu [10]
*Serratia marcescens*		
CB40	Encapsulated clinical isolate; Cm^r^	This study
CB47	Encapsulated clinical isolate; Cm^r^	This study
*Acinetobacter baumannii*		
AB08	Encapsulated clinical isolate; Cm^r^	This study
AB23	Encapsulated clinical isolate; Cm^r^	This study
*Klebsiella pneumoniae* CG354	Encapsulated clinical isolate; Cm^r^	This study
Plasmids		
p3ZLux4	ColE1-typed replicon and *lux* operon; Ap^r^; Km^r^;	This study
pKD46Gm	A temperature-sensitive plasmid of λ Red mutagenesis vector; Gm^r^	Doublet *et al*. [11]
pSE-Lux1	46.3-kb conjugative plasmid with IncX- and ColE1-typed replicon and *lux* operon; Ap^r^, Km^r^;	Chen *et al.*[7]

### Mutagenesis in *S*. Typhimurium

Invasion effector gene *sipB* gene of *Salmonella* pathogenicity island 1 (SPI-1) was chosen to construct less virulence mutants of *S.* Typhimurium via the phage Lambda Red recombinase expression system [12],[13]. The primer sequences were designed according to the sequences of *sipB* of *S*. Typhimurium LT2 (accession number NC_003197) and *cat* (a chloramphenicol acetyl transferase-encoding gene) of pKD3 [13]. The forward sequence SipB-Cm-F (5’-TGGAGTCTCG TCTGGCGGTA TGGCAGGCGA TGATTGAGTC cgcctacctg tgacggaaga-3’) and the reverse sequence SipB-Cm-R (5’-GCTGCGGTAT TCGTGACTTC CATGCCCAAC GCCACTTTAT ccctgccact catcgcagta-3’) were generated a 932-bp PCR amplicon, where the *sipB* gene portion is presented in capital letter and the *cat* portion in lower case. *S.* Typhimurium strains OU5045 and OU5046 had been previously transformed with a Lambda Red recombinases-carrying pKD46Gm by electroporation [10],[13]. The *S.* Typhimurium *sipB*-deleted mutants were named OU5045△*sipB* and OU5046△*sipB*.

### Plasmid conjugation and stability

The conjugation test was conducted with a slight modification of methods described elsewhere [7]. *E. coli* TOP10 and *S.* Typhimurium OU5046 carrying pSE-Lux1 played as donors, the drug-susceptible recipient bacteria, including *A. baumannii* strains AB08 and AB23, *E. coli* TOP10, *K. pneumoniae* strain CG354, *S.* Typhimurium OU5045 and OU5046, and *S.* Choleraesuis OU7085, and *Serratia marcescens* strains CB40 and CB47, were electroporated with gentamicin-resistant plasmid the pKD46Gm [11]. Overnight donor and recipient bacteria were 10-fold diluted with LB broth and then co-cultured at 37°C without agitation for 16 hrs. Transconjugants and recipient bacteria were counted by plating bacteria on LB agar containing appropriate antibiotics. The conjugation efficiency of each recipient bacterium was determined by dividing the number of transconjugants by the total of recipients.

Plasmid stability was determined by methods as described elsewhere [14]. Bacteria were cultured in LB broth medium without antibiotic and subcultured twice a day for a period of four days (D0-D4) in a 1000-fold dilution (approximate 80 generations in four-day period). The number of bacteria (CFU) was counted on agar media with or without kanamycin and ampicillin for both pSE-Lux1 and p3ZLux4 (Table 1) to select the bacteria with or without the plasmid. The plasmid stability was determined by the ratio of the bacterial survival number on selective media to the total bacterial number on non-selective media [5],[6].

### Evaluation of bacterial infection in mice

Animal test of bacterial infection in Balb/c mice was approved by the Institutional Animal Care and Use Committee (CGU11-164) of Chang Gung University, Taoyuan, Taiwan. Overnight-grown bacteria were washed and re-suspended with phosphate buffered saline (PBS) and then was used to challenge the mice via intravenous (IV) injection and oral administration [15],[16]. The mice were anesthetized by gas anesthesia (3% isoflurane), and then imaged by the *in vivo* imaging system *IVIS*® *100 Series* (Xenogen) in accordance with the guidelines of Guide for the Care and Use of Laboratory Animals [17]. After animal experiments or once illness appearances, mice were euthanized according to the previous guidelines.

For a long-term bioluminescence test of a systemic bacterial infection using the pSE-Lux1-carrying bacteria in mice, the mice (n = 3) were intravenously injected with 10^7^ CFU of *K. pneumoniae* CG354 (a strain non-pathogenic to mice). The mice were tested for 6 days without selective pressures, and then were euthanized by breaking the neck while still anesthetized.

For the evaluation of gastrointestinal infection of *Salmonella* in mouse, the mice were orally gavaged with 10% sodium bicarbonate to neutralize stomach acid for 30 minutes prior to *Salmonella* challenge. The mice were separated into four groups (n = 5 per group) and each mouse were then orally gavaged with 10^9^ CFU *S.* Typhimurium. The strains were wild type OU5045, virulence plasmid-less OU5046, and *sipB*-deletion mutant strains OU5045△*sipB* and OU5046△*sipB*. The mice were tested without selective pressures for four hours to strains OU5045 and OU5046, and for two days to mutants due to the restriction of mouse illness appearance thereafter. The anatomic organs of the tested mice were analyzed in parallel for IVIS imaging.

### Statistical analysis

Pair-wise comparison in one-way ANOVA (ANalyses Of VAriance between groups) test was performed using the software program of Statistical Product And Service Solutions (SPPS 12.0), and followed by a Tukey’s HSD (Honestly Significant Difference) test to determine the significance of difference between p3ZLux4 and pSE-Lux1 in the tests of plasmid stabilities.

## Results

### Plasmid pSE-Lux1-mediated conjugation tests between Gram-negative bacteria

The conjugation results showed that plasmid pSE-Lux1 enabled to be transferred into all tested Gram-negative bacteria, even into the clinical mucoid (encapsulated) *A. baumannii*, *K. pneumoniae*, and *S. marcescens* (Table 2). The highest conjugation efficiencies of pSE-Lux1 in the donor *E. coli* TOP10 were 7.5 ± 2.4 × 10^−3^ and 1.2 ± 0.4 × 10^−3^ for *S.* Choleraesuis SC7085 and *S. marcescens* CB47. Additionally, conjugation efficiencies were observed higher in the same species than between species; for examples, *E. coli-*to*-E. coli* versus *E. coli*-to-*S.* Typhimurium (1.8 ± 1.6 × 10^−4^ vs. 1.0 ± 0.8 × 10^−6^), and conversely, *S.* Typhimurium*-*to*-S.* Typhimurium versus *S.* Typhimurium*-*to-*E. coli* (1.4 ± 1.6 × 10^−3^ vs. 2.2 ± 1.1 × 10^−6^)*.* Furthermore, conjugation efficiency was strain-dependent. Transfer of pSE-Lux1 differed between two recipient *S. marcescens* strains CB47 and CB40 with a respective value of 1.2 ± 0.4 × 10^−3^ and 5.9 ± 5.1 × 10^−7^ (Table 2).

**Table 2:** Conjugation efficiency of pSE-Kux1 among various Gram-negative species

**Donor bacteria**	**Recipient bacteria**	**Conjugation efficiency**^#^
*E. coli*/pSE-Lux1	*A. baumannii* AB08	1.2 ± 0.6 × 10^−7^
	*A. baumannii* AB23	8.1 ± 3.8 × 10^−6^
	*E. coli* TOP10	1.8 ± 1.6 × 10^−4^
	*K. pneumoniae* CG354	1.9 ± 1.3 × 10^−6^
	*S.* Choleraesuis SC7085	7.5 ± 2.4 × 10^−3^
	*S.* Typhimurium OU5045	1.0 ± 0.8 × 10^−6^
	*Serratia marcescens* CB40	5.9 ± 5.1 × 10^−7^
	*Serratia marcescens* CB47	1.2 ± 0.4 × 10^−3^
*S.* Typhimurium OU5046/pSE-Lux1	*E. coli* TOP10	2.2 ± 1.1 × 10^−6^
	*K. pneumoniae* CG354	8.4 ± 4.6 × 10^−7^
	*S.* Typhimurium OU5045	1.4 ± 1.6 × 10^−3^

^#^Conjugation efficiencies were determined from three individual tests.

### Difference in plasmid stability of pSE-Lux1 and p3ZLux4 within various bacteria

**Figure 1:**
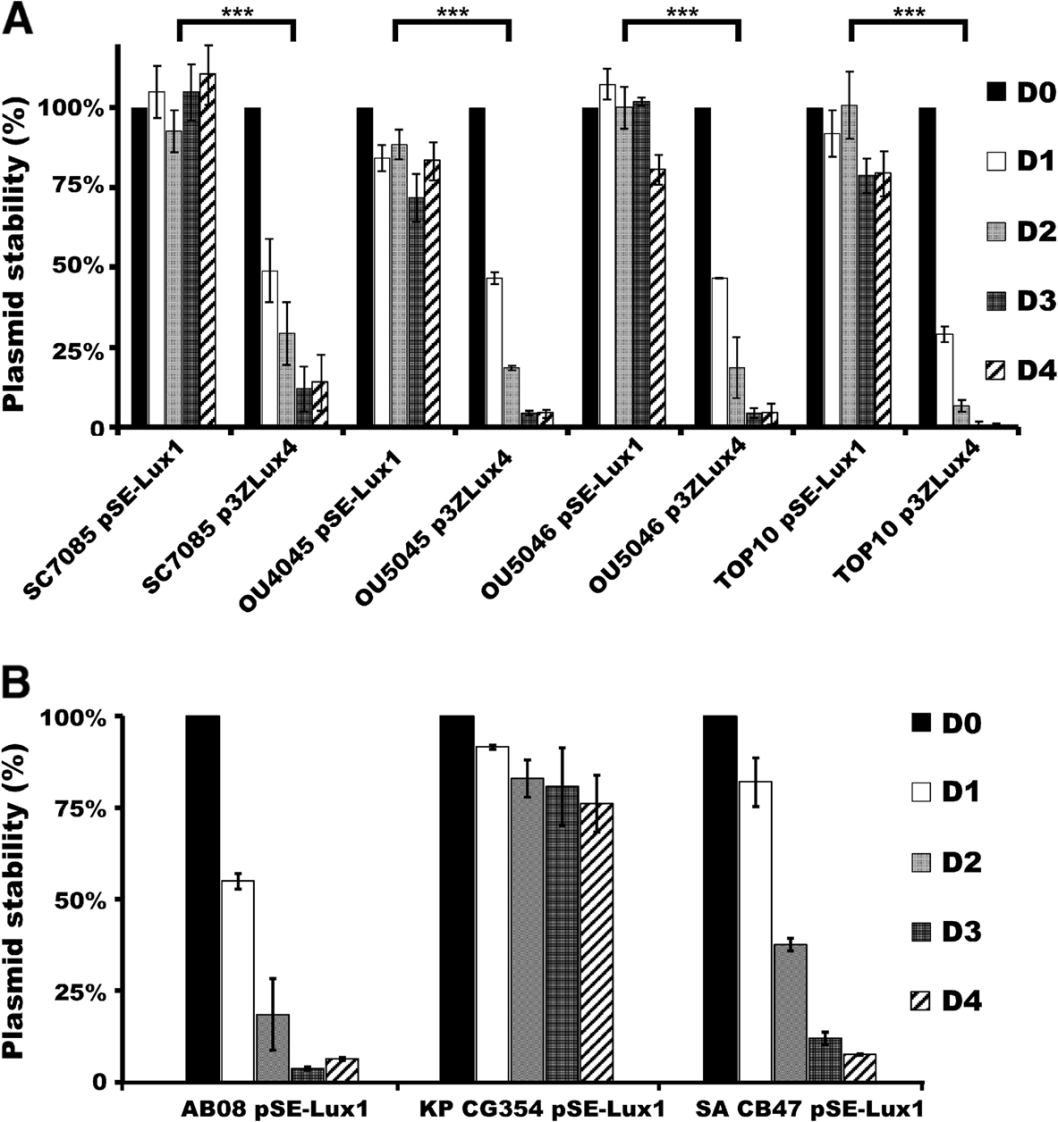
**Plasmid stabilities. (A)** The plasmid stabilities of pSE-Lux1 and p3ZLux4 are compared within various bacteria for four days. The tested bacteria include *S.* Choleraesuis SC7085, *S.* Typhimurium OU5045 and OU5046, and *E. coli* TOP10. ***: significance with *P* value less than 0.001 (*P <* 0.001). **(B)** The plasmid stabilities of pSE-Lux1 within the encapsulated bacteria, including *Acinetobacter baumannii* (AB) AB08, *K. pneumoniae* (KP) CG354, and *Serratia marcescens* (SA) CB47, were tested. D0 represents the day zero when the stability test was initiated, and its value was calibrated as 100%, proportionally compared to those analyzed at the other time periods, including D1, D2, D3, and D4, which are denoted the day one, the day two, the day three, and the day four, respectively.

Plasmid stability of pSE-Lux1 and p3ZLux4 differed among non-capsulated *S.* Choleraesuis SC7085, *S.* Typhimurium OU4045 and OU5046, and *E. coli* TOP10 (Figure 1A). pSE-Lux1 maintained with at least 75% of stability at four-day duration (around 80 generations) in all four bacteria; the highest stability was observed in *S.* Choleraesuis SC7085 (almost 100%). In contrast, p3ZLux4 was less stable (<14%) than pSE-Lux1 in all tested bacteria with significant difference between these two plasmids (*P* < 0.001). Coincidently, p3ZLux4 was the most stable in *S.* Choleraesuis SC7085 than other tested bacteria. In capsulated *K. pneumoniae* CG354, *A. baumannii* AB08 and *S. marcescens*, p3ZLux4 DNA was failed to transform into these bacteria by electroporation. Therefore, we only evaluated the stability of pSE-Lux1. The stability of pSE-Lux1 was higher in *K. pneumoniae* CG354 than in *A. baumannii* AB08 and *S. marcescens* CB47 (Figure 1B).

### Long-term monitoring of bioluminescence bacterium in mice with a systemic bacterial infection

**Figure 2:**
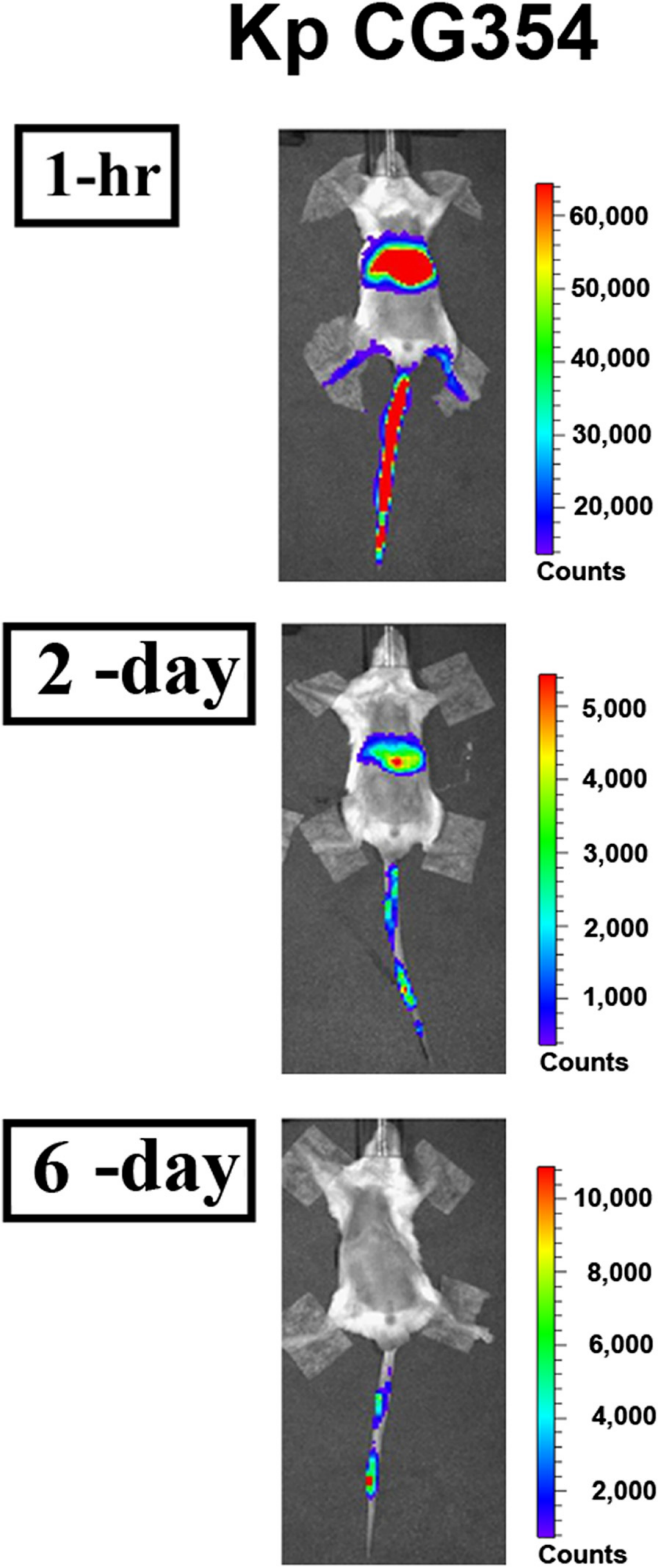
**A long-term bioluminescence test in mice with a systemic bacterial infection.** The pSE-Lux1-carrying strain CG354 of *K. pneumoniae* was intravenously injected into Balb/c mice. The bioluminescence signals from the mice were monitored via an IVIS system during the periods from one hour to six days post-systemic bacterial inoculation. The photos were taken with 0.5-second exposures. The color bar is indicated beside each IVIS image.

Non-pathogenic *K. pneumoniae* strain CG354 carrying pSE-Lux1 was used. The bioluminescence signals were clearly observed from the mice without selective pressures (Figure 2). Although the signal was gradually weakened within six-day period, the signal could still be clearly detected at the portion of mouse tail at the sixth day.

### Bacterial distributions of the route of gastrointestinal infection in mice

**Figure 3:**
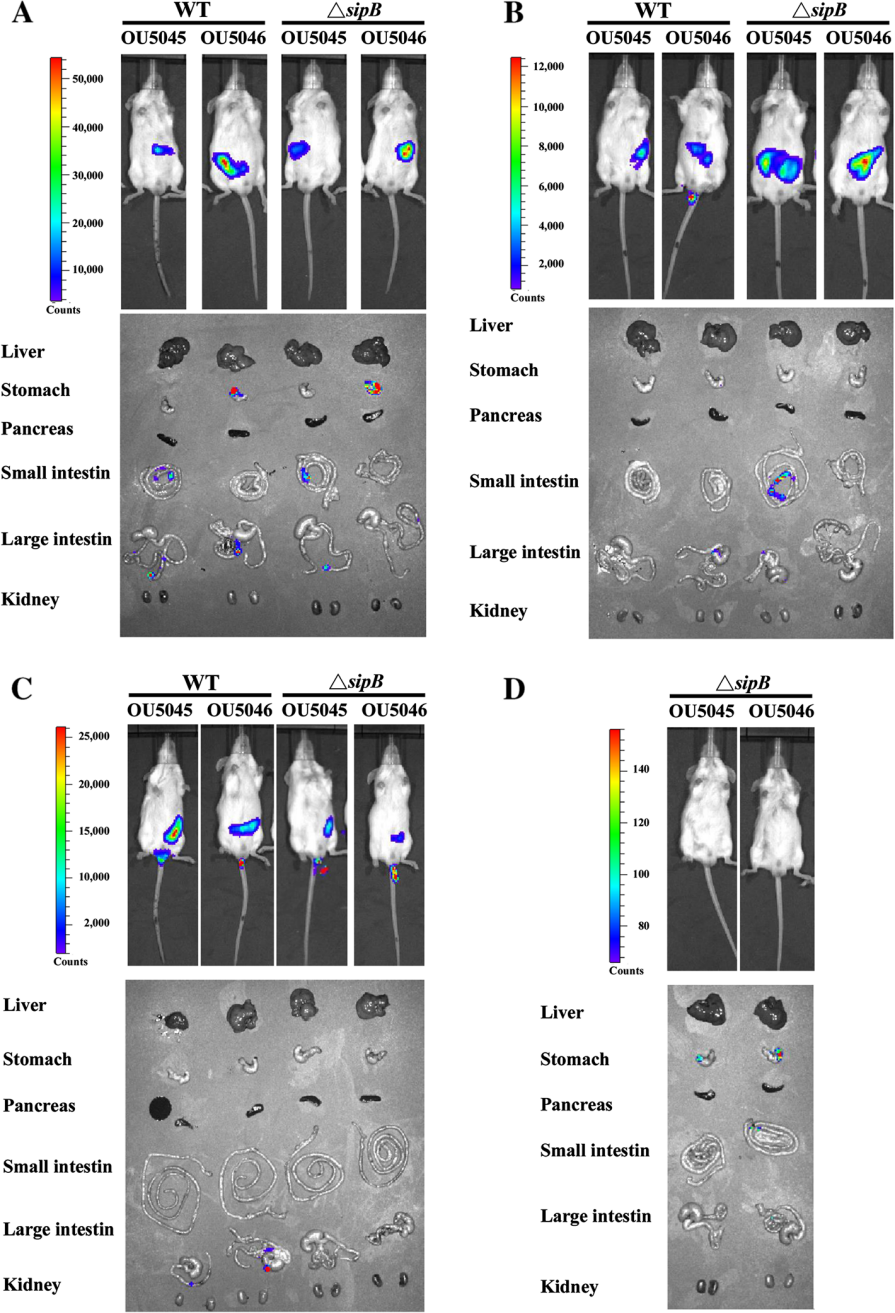
**Dynamic distribution of*Salmonella*in mouse gastrointestinal tracts.** The wild type (WT, including OU5045 and OU5046 strains) and *sipB*-deletion mutants (including OU5045△*sipB* and OU5046△*sipB* strains) of *S.* Typhimurium were orally administrated into Balb/c mice. The bioluminescence signals from living mice as well as from their anatomic organs in parallel (lower part of each panel) were monitored at the first- **(A)**, second- **(B)**, fourth- **(C)**, and 48^th^-hour **(D)** periods after *Salmonella* inoculations. The pictures were taken with 3-minute exposures via IVIS. The color bar is indicated beside each IVIS image.

Four *S.* Typhimurium isogenic strains OU5045, OU5046, and *sipB*-deletion mutant strains OU5045△*sipB* and OU5046△*sipB* were transformed with pSE-Lux1. The bioluminescence signals from the mice demonstrated that most bioluminescence *Salmonella* travelled rapidly from stomach, small intestine, and large intestine to anal within four hours, and the bacteria were mostly shed at the 48^th^ hour post-bacterial inoculation (Figure 3).

No significant difference in bioluminescence patterns was observed among the four isogenic strains within gastrointestinal tracts of mice. The bioluminescence signals were matched the amount of living bacteria from the anatomic organs. However, the periods of illness appearance differed between the *sipB*-normal and the *sipB*-deletion strains (four hours vs. two days post-*Salmonella* inoculations). Noteworthily, the bioluminescence signals present in the portion of stomach organs for the virulence plasmid-less OU5046, but not in its wild-type OU5045.

## Discussion

The green fluorescence protein (GFP)-associated fluorescence method and the luciferase-associated bioluminescence method are the two major methods for IVIS imaging system in animal models. Compared to GFP fluorescence, two advantages of the *luxABCDE-*mediated bioluminescence method are A) only metabolically active and living bacteria may present light, but the dormant or dead bacteria may not, or weakly, because of their little production of aldehyde substrates for luciferase reactions; B) bioluminescence background of this system is low in animal models [18]. However, the GFP fluorescence method has higher sensitivity than bioluminescence methods in IVIS system and this advantage is only available to superficial organs less than 6 mm depth from the surface of test animals, or fluorescence signals would be faded [19]. Moreover, auto-fluorescence backgrounds emitted from animals are high [3]. To overcome auto-fluorescence, the test animals should get starved for 3 to 24 hours prior to fluorescence imaging, because diets may cause significant auto-fluorescence [20]. The precaution by starvation may limit the application of GFP-associated methods in IVIS imaging systems, particularly at the detection sites close to gastrointestinal tracts.

First bioluminescence image of the *lux* operon was developed to study the pathogenesis of *S.* Typhimurium in C57BL/6 or BALB/c mice using an artificial plasmid that contains this operon from *Photorhabdus luminescens*[21]. However, the replication origin of this plasmid was derived from the ColE1 replicon and was unstable without antibiotic pressure. Therefore, three stable photonic plasmids pCGLS-1 (carrying ColE1 replicon), pAK1-lux (carrying pBBR1 replicon) and pXEN-1 (carrying both pC194 and ColE1 replicons) were constructed for the stability in *S.* Typhimurium [1]. In this study, a pSE34-based pSE-Lux1 showed better plasmid stability than ColE1 replicon-based p3ZLux4 in *E. coli*, *K. pneumoniae*, and *S. enterica*. This is probably because pSE-Lux1 carries important genetic elements, including ColE1 and IncX replicons, conjugation-associated *pil* operon, and plasmid maintenance-associated genes *pir*, *parG*, *parF*, *stbD*, and *stbE*[7]*.* Some other bioluminescence systems (such as *fluc*, g*luc*, or *rluc*) may be more appropriate than *luxABCDE* in various bacteria [2],[3],[22]. However, firefly Fluc and Gaussia Gluc require the intravenous addition of substrate luciferins for bioluminescence catalyzation *in vivo,* therefore, it only remains relatively as short as 30 minutes when the peak of bioluminescence signal reaches a plateau [23].

The conjugation efficiencies differed between bacterial species probably due to difference in enzymatic restriction and modification system. However, the two recipient *S. marcescens* strain CB40 and CB47 showed dramatically different conjugation efficiencies and this difference may be attributed to the different genomic background or bacterial capsule. With regard to the bioluminescence patterns of *K. pneumoniae* mucoid strain CG354 for a systemic bacterial infection in this study, the gradual reduction of bioluminescence signals over the time indicated that strain CG354 was non-pathogenic to mice. Similar to strain CG354, strain IA565 of *K. pneumoniae,* a human clinical isolate, is known to be non-pathogenic to mice [24]. However, survey of International *Klebsiella* Study Group reported that 69% mucoid clinical strains are pathogenic to murine [16].

In the bioluminescence patterns of anatomic gastrointestinal organs of mice, pSE-Lux1-carrying OU5046 strain produced more signals in the stomach than by the pSE-Lux1-carrying OU5045 strain were found. It is likely because the more virulent strain caused more severe inflammatory diarrhea to mice, more *Salmonella* shed away from stomach and other gastrointestinal organs, and therefore, the less virulent △*sipB* mutant remained more in gastrointestinal tracts. Although *Salmonella* are known to enable survive in the acidic environment of stomach through the induction of the acid tolerance response, it still remains unclear why *Salmonella* can colonize in the stomach; however, its colonization may explain why *Salmonella* can cause stomach cramps in humans [25],[26].

Bioluminescence-related publications have been increasing in application to study in the area of pathogenicity, tumorigenicity, biofilm, and dermatology [27]–[30]. Moreover, the bioluminescence vehicle can be genetically engineered to carry some other potential exogenous genes, such as anticancer agents for therapeutic purposes [31].

## Conclusion

In this study, a novel stable and conjugative bioluminescence pSE-Lux1 vehicle system available in a broad range of bacteria, even for encapsulated bacteria, is well developed and applied to investigate the infection route of pSE-Lux1-carrying bacteria in living mice

## Competing interest

No conflict of interest declared.

## Authors’ contribution

Study design and data collection: HY-K, Chen C-L, WC-H and Chiu C-H carried out the study design; HY-K, Chen C-L and Chiu C-H carried out the molecular data analysis. Chen C-L and Chiu C.-H carried out the experimental data interpretation. HY-K and Chen C-L participated in the sequence alignment and drafted the manuscript. Chu C, Chen C-L and Chiu C-H refined the manuscript. All authors read and approved the final manuscript.
